# Case report: Cystic fibrosis with kwashiorkor: A rare presentation in the era of universal newborn screening

**DOI:** 10.3389/fped.2022.1083155

**Published:** 2023-01-06

**Authors:** Annemarie G. Wolfe, Stephanie P. Gilley, Stephanie W. Waldrop, Christina Olson, Emma Harding, Kaitlin Widmer, Lindsey B. Gumer, Matthew Haemer, Jordana E. Hoppe

**Affiliations:** Department of Pediatrics, University of Colorado Denver Anschutz Medical Campus, Aurora, CO, United States

**Keywords:** cystic fibrosis - CF, kwashiorkor, newborn screening (NBS), pancreatic insufficiency, missed diagnosis

## Abstract

**Background:**

Universal newborn screening changed the way medical providers think about the presentation of cystic fibrosis (CF). Before implementation of universal screening, it was common for children with CF to present with failure to thrive, nutritional deficiencies, and recurrent infections. Now, nearly all cases of CF are diagnosed by newborn screening shortly after birth before significant symptoms develop. Therefore, providers often do not consider this illness in the setting of a normal newborn screen. Newborn screening significantly decreases the risk of complications in early childhood, yet definitive testing should be pursued if a patient with negative newborn screening presents with symptoms consistent with CF, including severe failure to thrive, metabolic alkalosis due to significant salt losses, or recurrent respiratory infections.

**Case presentation:**

We present a case of a 6-month-old infant male with kwashiorkor, severe edema, multiple vitamin deficiencies, hematemesis secondary to coagulopathy, and diffuse erythematous rash, all secondary to severe pancreatic insufficiency. His first newborn screen had an immunoreactive trypsinogen (IRT) value below the state cut-off value, so additional testing was not performed, and his growth trajectory appeared reassuring. He was ultimately diagnosed with CF by genetic testing and confirmatory sweat chloride testing, in the setting of his parents being known CF carriers and his severe presentation being clinically consistent with CF. Acutely, management with supplemental albumin, furosemide, potassium, and vitamin K was initiated to correct the presenting hypoalbuminemia, edema, and coagulopathy. Later, pancreatic enzyme supplementation and additional vitamins and minerals were added to manage ongoing deficiencies from pancreatic insufficiency. With appropriate treatment, his vitamin deficiencies and edema resolved, and his growth improved.

**Conclusion:**

Due to universal newborn screening, symptomatic presentation of CF is rare and presentation with kwashiorkor is extremely rare in resource-rich communities. The diagnosis of CF was delayed in our patient because of a normal newborn screen and falsely reassuring growth, which after diagnosis was determined to be secondary to severe edematous malnutrition. This case highlights that newborn screening is a useful but imperfect tool. Clinicians should continue to have suspicion for CF in the right clinical context, even in the setting of normal newborn screen results.

## Introduction

Newborn screening for cystic fibrosis (CF) in the United States was adopted in the early 1980s and became standard in all fifty states by 2010 (1, 2). Prior to the implementation of universal newborn screening and in countries where newborn screening is not performed, many children were diagnosed with CF after developing symptoms including malnutrition, growth faltering, chronic cough, recurrent respiratory infections/pneumonias, rectal prolapse, and/or electrolyte and other nutritional abnormalities (1). Due to the success of newborn screening, clinicians in high resource settings have never seen a child present with symptomatic CF and may not consider CF when these symptoms occur.

Early diagnosis of CF has been shown to improve outcomes due to optimized nutrition and targeted interventions (3). Numerous studies over the years continually reconfirm that patients do better when diagnosis happens earlier (3–5). Improved growth, presumably secondary to early initiation of pancreatic enzyme replacement therapy and other vitamin supplementation, leads to better pulmonary function throughout life as well as improved neurological outcomes into adolescence. Malnutrition in children with CF tends to lead to increased lung disease (3). Seventy percent of infants that were symptomatic at presentation had more hospitalizations in the first year of life and more complications, including growth faltering, positive *Pseudomonas aeruginosa* culture results, and electrolyte abnormalities when compared to patients diagnosed prenatally or *via* newborn screening (6, 7). Decreased chronic infection with *Pseudomonas aeruginosa* has also been observed since implementation of universal newborn screening (1) which leads to improved lung function.

**Figure 1:**
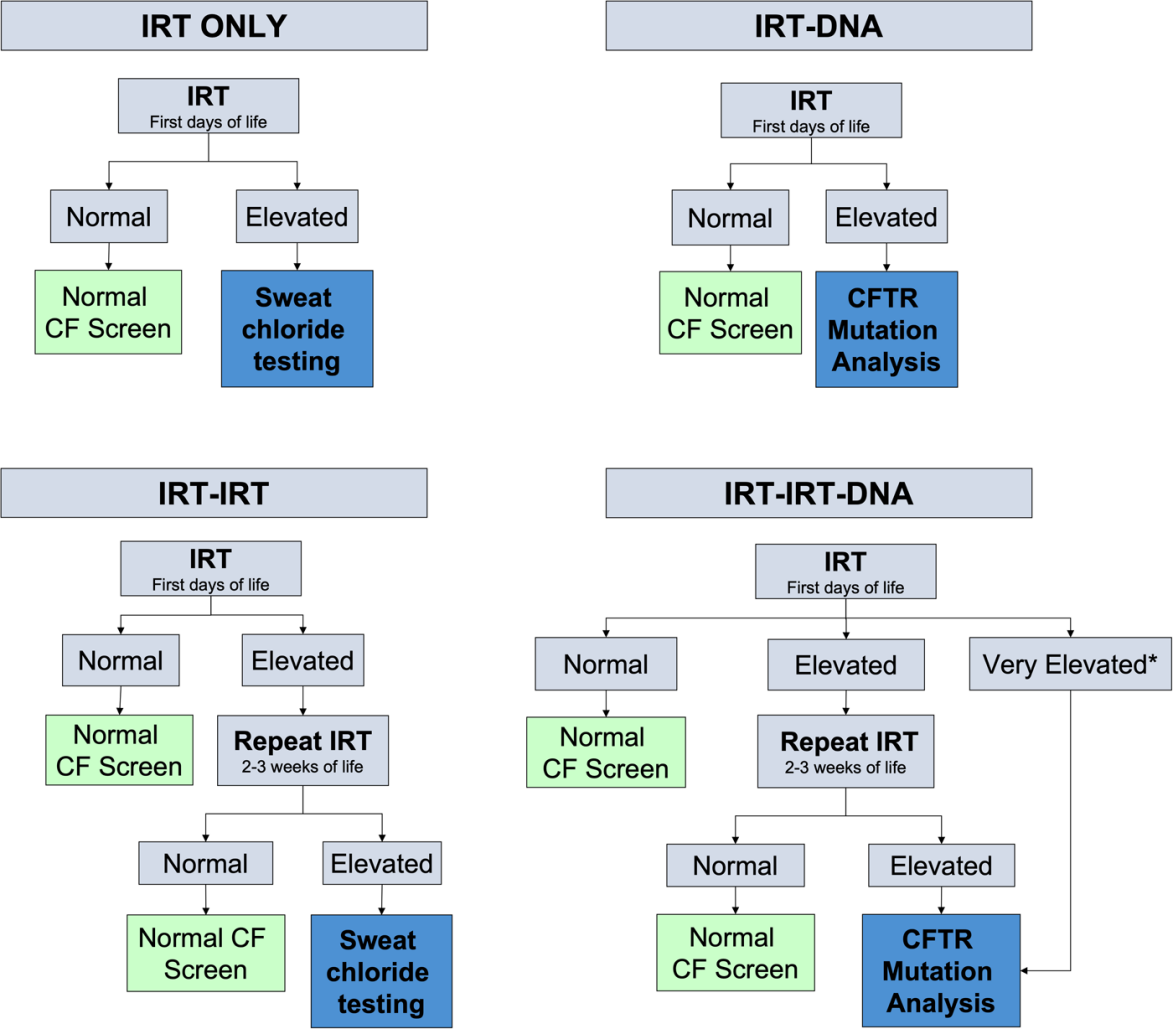
Different algorithms for newborn screening processes measuring newborn IRT levels in blood spot testing. Levels for normal or elevated also vary depending on the state. *Some states have automatic CFTR mutation analysis if the IRT level is above a certain threshold.

Newborn screening is performed with measurements of immunoreactive trypsinogen (IRT) and genetic testing but testing protocols vary from state to state (8) (Figure 1). IRT is a pancreatic precursor enzyme that is elevated in patients with CF due to pancreatic duct blockage (9). An elevated IRT is not specific to CF and can be elevated in the absence of CF, particularly when infants are born premature, have low Apgar scores, or experience perinatal stress (3). In all states, an IRT level above a certain threshold is indicative of a positive screen; however, the cutoff varies and is not specified in the American College of Medical Genetics and Genomics guidelines (10), allowing states to set their own threshold. Different procedures for newborn screening for CF include IRT-only, IRT-IRT, IRT-DNA, and IRT-IRT-DNA (8, 9, 11). IRT-only states measure IRT levels in blood samples from newborns collected around 2 weeks of life and if elevated, proceed directly to sweat testing. In IRT-IRT states, the first IRT sample is collected in the first few days of life and if abnormal, a repeat IRT will be collected. If the repeat IRT remains elevated, the next step is sweat chloride testing (11). In IRT-DNA states, the sample is typically collected in the first days of life and the sample is reflexively sent for DNA testing if the IRT level is above a particular threshold. In IRT-IRT-DNA states, the first sample is collected within 24 h of life, and if negative, no further testing is done. If the initial IRT is elevated in these states, IRT testing will be repeated on the second screen, sent around 2 weeks of life. If the IRT remains elevated, then reflex testing for mutations in the cystic fibrosis transmembrane conductance regulator (CFTR) gene occurs, either with a panel of common genetic variants seen in that state or full genetic sequencing (12). For all states, patients flagged with abnormal newborn screens have a protocol for confirmatory testing with sweat chloride testing, which remains the gold standard for diagnosing cystic fibrosis (9). All states have varying, non-zero false negative rates, meaning that all states will intermittently miss true cases of CF on the screen. It is estimated that newborn screening with any IRT process identifies approximately 95%–99% of newborns with CF (13).

## Case presentation

**Figure 2:**
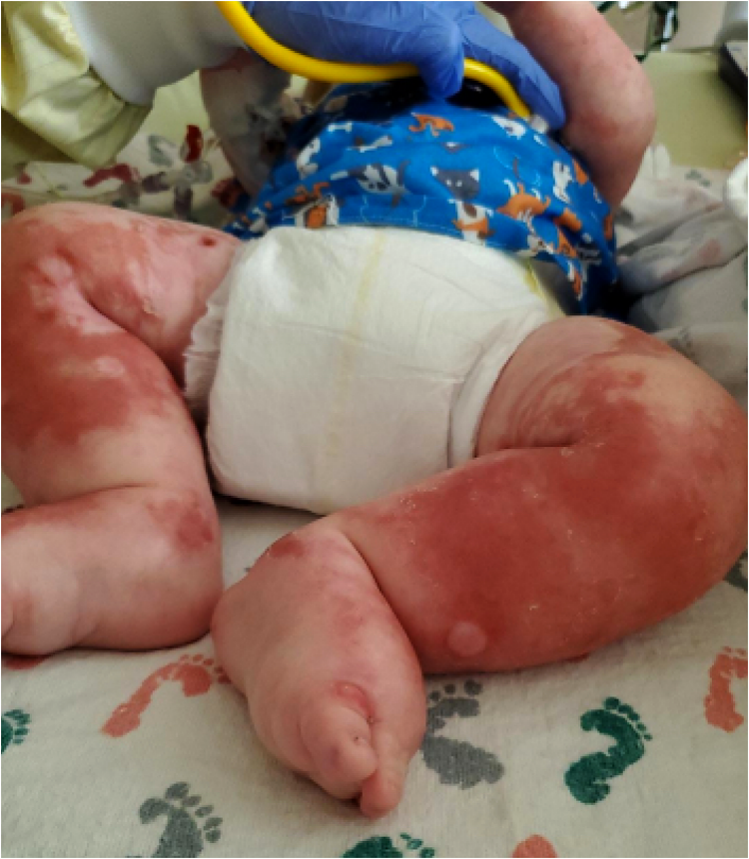
Patient's lower extremities on presentation demonstrating edema and diffuse rash with scaly plaques and bullous areas.

A 6-month-old Caucasian male presented with 2 weeks of fussiness, fever, and decreased activity and one episode of hematochezia and hematemesis in the setting of 1 month of a progressively worsening rash that started in his diaper area and spread to the extremities, neck, and trunk (Figure 2). There was no change in his rash with emollients or topical steroid use.

**Figure 3:**
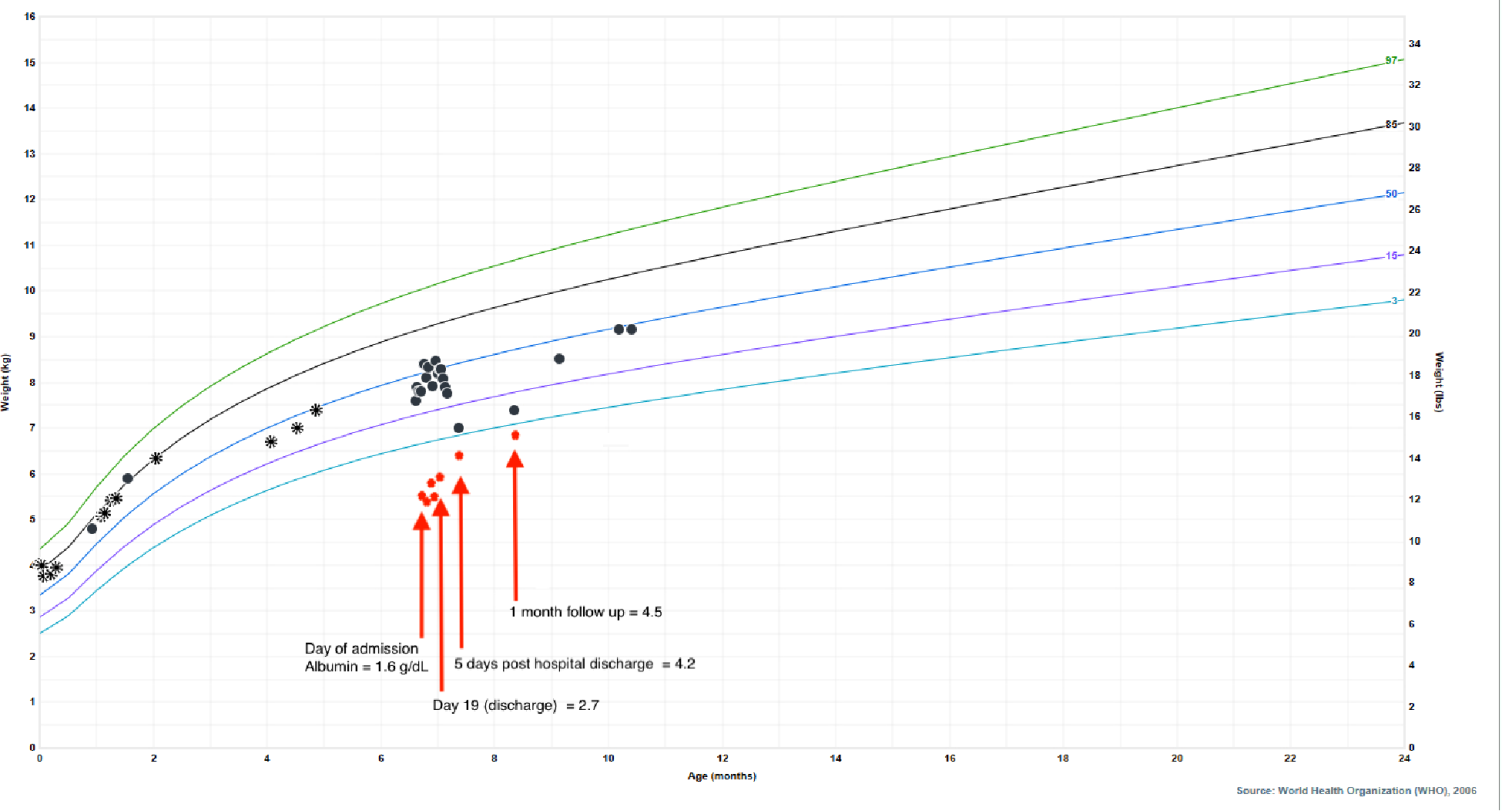
Patient's growth chart for weight from birth to present day with superimposed albumin values to highlight the impact of hypoalbuminemia on his falsely reassuring growth curve. Circle points indicate hospital system measurements, asterisk points indicate outside of hospital measurements. Albumin normal lab value range: 3.4–4.2 g/dl.

He was born full term without complications, passed meconium in the first 24 h of life, and had a normal newborn screen. His initial IRT was 59.3 ng/ml (normal <60 ng/ml), so no further testing was performed according to the state protocol. Parents were both identified as CF carriers during the pregnancy. His weight dropped from the 85th percentile at 2 months of age to the 33rd percentile at 4 months of age which coincided with a transition from exclusive direct breastfeeding to mostly bottle feeding of expressed breast milk. Weight gain from that point forward was consistent around the 35th percentile. His length, however, dropped from the 73rd percentile at 2 months of age to less than 3rd percentile at presentation at 6 months of age (Figure 3). He was taking adequate volumes of breastmilk on demand about every 3–4 h with occasional reflux episodes. He was taking minimal solid foods at the time of admission. Vitamin D supplementation was inconsistent. His stooling pattern was reported as 3–4 times per day, soft consistency without malodor, discoloration, or oil droplets except for one episode of bright red blood per rectum immediately prior to admission. His mother reported she had concerns about constipation due to crying and fussiness with stooling. He had no respiratory symptoms such as cough or history of pneumonia.

Physical examination on admission was remarkable for generalized edema (facial, periorbital, peripheral) with a diffuse, reddish-brown in color, serpiginous, rash with scaly plaques at his extremities and perineal area (Figure 2). The rash was most prominent in the flexural surfaces with darker purple appearance in the intertriginous areas. There was an approximately three centimeter left inguinal mass with overlying purpura. His breathing was comfortable without any adventitious breath sounds, and his abdomen was soft, nontender, and without appreciable organomegaly, though this was difficult to assess due to his anasarca. His hair was thin and reddish in color.

Labs were notable for coagulopathy, hypoalbuminemia, hyponatremia, elevated liver enzymes, elevated ferritin, elevated lactate dehydrogenase (LDH), elevated c-reactive protein, decreased 25-hydroxy vitamin D level, decreased retinol/vitamin A level, decreased vitamin E alpha tocopherol level, and decreased zinc level (Table 1). Stool was positive for occult blood. Due to multiple fat-soluble vitamin deficiencies, fecal pancreatic elastase was assessed and was low. His potassium was initially normal, but quickly dropped to 2.1 mmol/L on the second day of his hospitalization. He also tested positive for SARS-CoV-2 on admission. Of note, his father had tested positive for SARS-CoV-2 approximately 1 week prior to admission.

**Table 1:** Labs values at the time of presentation with normal reference ranges.

Lab name	Value	Normal range
Sodium	130 mmol/L	134–143 mmol/L
Potassium	2.1 mmol/L	3.4–4.7 mmol/L
Albumin	1.6 g/dl	3.4–4.2 g/dl
Alanine transaminase (ALT)	186 U/L	12–45 U/L
Aspartate aminotransferase (AST)	159 U/L	20–60 U/L
Gamma-glutamyl transferase (GGT)	306 U/L	5–16 U/L
Ferritin	1,200 ng/ml	10–95 ng/ml
LDH	1,532 U/L	400–1,230 U/L
C-reactive protein	2.6 mg/dl	0.0–0.9 mg/dl
Retinol/vitamin A	6 µg/dl	19–77 µg/dl
25-hydroxy vitamin D	9.5 ng/ml	30–96 ng/ml
Vitamin E alpha tocopherol	1.9 µg/ml	3.8–20.3 µg/ml
PTT	75 s	22–37 s
INR	>9	
Zinc	0.25 mcg/ml	0.60–1.2 mcg/ml
Fecal pancreatic elastase	40 mcg/g	>200 mcg/g

He was admitted and diagnosed with kwashiorkor with prompt and cautious initiation of treatment for severe malnutrition, electrolyte derangements, and coagulopathy. Consultants involved included gastroenterology, hepatology, genetics/metabolism, allergy/immunology, cardiology, hematology, nutrition physicians, pulmonology, infectious disease, and dermatology. He received intravenous vitamin K and was initially started on continuous nasogastric feeds with breastmilk fortified with a high-protein extensively hydrolyzed formula. He received intravenous antibiotics for inguinal lymphadenopathy and cellulitis with intravenous antibiotics. Based on his very low fecal elastase measurements without diarrhea and his persistent edema and electrolyte derangements, he was started on empiric pancreatic enzymes on hospital day 10 pending a unifying diagnosis. Given the severity of his presentation and the broad differential diagnosis, rapid whole exome sequencing was pursued which identified two mutations in CFTR: F508del and 1717-1G->A, leading to a diagnosis of CF. No other genetic mutations were identified. A sweat chloride test was not obtained during his hospitalization due to the severity of his rash and electrolyte derangements but was performed about 1 month after hospitalization. One month after discharge, his sweat chloride levels were elevated at 86 and 90 mmol/L (normal <60 mmol/L) on his right and left arms respectively, confirming a diagnosis of CF.

Ultimately, his presenting diagnoses of kwashiorkor and coagulopathy were considered secondary to chronic CF-associated pancreatic insufficiency and malabsorption. Kwashiorkor explained his edema, hypoalbuminemia, elevated liver enzymes, hypoglycemia, malabsorption, thin and reddish hair, and rash. He demonstrated improvement with continuous nasogastric feeds of fortified breastmilk, supplemental fat-soluble vitamins, pancreatic enzymes, and electrolyte replacements. He was discharged home on 24 kcal/oz fortified oral and nasogastric feeds, DEKA vitamins, supplemental vitamin D3 (cholecalciferol) and zinc, and pancreatic enzymes. Additionally, he was started on twice daily respiratory treatments with albuterol and chest physiotherapy. After diuresis, his weight dropped to the 4th percentile, which correlated with normalization of his albumin (Figure 3), indicating that the appearance of his reassuring growth prior to presentation was weight gain secondary to edema and not true growth. After interventions, his weight appropriately increased and his stunted length began to normalize, signaling improved absorption and adequate nutrition. Growth measurements 5 months after hospitalization demonstrate significant recovery with weight at the 45th percentile, length at the 11th percentile, and weight-for-length at the 75th percentile.

## Discussion and conclusion

Prior to newborn screening for CF, the average age at diagnosis was 2.9 years (in 1995) and children typically presented with malnutrition and growth faltering (14). Additionally, a symptomatic presentation could include dehydration, steatorrhea or abnormal stools, electrolyte abnormalities, recurrent respiratory infections and sinus disease, or meconium ileus at birth. Electrolyte derangements most commonly included hyponatremic, hypochloremic, and hypokalemic metabolic alkalosis along with hypoproteinemia and edema (15). Fat soluble vitamin deficiencies were common due to pancreatic insufficiency. It is now well-known that CF outcomes are improved in patients who are diagnosed earlier in life (1, 14). In the early 1990s, Farrell et al. demonstrated that patients diagnosed earlier had significantly higher height and weight percentiles not only at the time of diagnosis, but also during the 10-year follow up period. They also found that patients diagnosed by newborn screening rather than symptoms had less severe lung disease during childhood (14). Today, most individuals with CF are diagnosed through newborn screening and subsequent confirmatory testing. The 2021 United States Cystic Fibrosis Foundation Registry Annual Data Report, as expected, reports that the majority of those diagnosed with CF in the first year of life are asymptomatic or mildly symptomatic due to newborn screening, DNA analysis, and prenatal testing (16). This means that the current generation of providers, particularly general practitioners, are not familiar with symptomatic presentation. Being unable to recognize symptomatic presentation of CF means that questionable symptoms may go unnoticed for longer in the setting of a normal newborn screen. Cases of CF presenting with kwashiorkor secondary to severe pancreatic insufficiency were documented prior to newborn screening and continue to be reported occasionally in low-resource communities (17–19). Kwashiorkor is a form of severe protein energy malnutrition seen in infants and young children and is more prevalent in low resource communities (20, 21). Manifestations can involve all body systems and are notable for peripheral pitting edema, marked muscle atrophy, depletion of fat stores, low weight-for-height, reduced mid-upper arm circumference, thin and dry skin, rash, dry hypopigmented hair, hepatomegaly from fatty liver infiltrates with abdominal distention, bradycardia, hypotension, and hypothermia (20). Other less commonly seen features include elevated liver enzymes, low serum concentrations of trace metals, and elevated ferritin concentrations (20). With implementation of universal newborn screening in the United States, CF presenting with kwashiorkor is now rarely seen, and as such, providers may not consider CF on the differential. According to the 2021 CF Registry Annual Data Report, no infants with CF have presented with edema at the time of diagnosis, highlighting the rarity of kwashiorkor (16). Our patient was receiving adequate intake by volume as assessed by parent report, leading to the concern that his kwashiorkor had an underlying cause rather than chronic inadequate intake.

Many patients with CF-related kwashiorkor have the characteristic kwashiorkor dermatosis: a rash with diffuse erythematous plaques, desquamation, and hyperpigmentation followed by peeling (18, 19, 22, 23). Similar appearing rashes can occur in patients with essential fatty acid deficiency (24) or severe zinc deficiency, particularly, acrodermatitis enteropathica, a rare genetic condition where intestinal zinc absorption is impaired (23, 25, 26). These diagnoses should also be considered, and a thorough dietary history is extremely important.

Newborn screening has resulted in identification of CF early in life, although not without false negatives. Children who are diagnosed with CF after a false negative newborn screen tend to have worse respiratory outcomes (7). Our patient's first newborn screen, obtained at 24 h of life, was reported as normal due to an IRT value of 59.3 ng/ml. He was born in an IRT-IRT-DNA state with a fixed cutoff of 60 ng/ml. Because his initial screen fell below the fixed cutoff, it was reported as normal, and so did not trigger repeat IRT or CFTR gene sequencing. His falsely normal newborn screen resulted in delayed consideration of CF as the cause for his rash, edema, and linear growth faltering. His substantial edema made his weight appear normal, providing further false reassurance due to what appeared to be adequate weight gain.

To attempt to limit the number of false negative cases and increase the sensitivity of screening, the cutoff values for newborn screening in our state have been adjusted (27). Instead of strict single cutoff value, the newborn screen process has been modified to have a floating cutoff calculated each day based on percentiles from daily IRT samples for the initial sample and fixed IRT cutoff of 50 ng/ml on the second sample. Variation in daily average IRT values have been observed with changes in seasons and reagents in the bloodspot kits, which supports the use of a floating cutoff (28). This also highlights that newborn screening programs should periodically re-evaluate their algorithm to identify areas for potential improvement. Areas for improvement include education, communication, and accuracy (2). Our case also emphasized the fact that the newborn screen is only a screen and not diagnostic; a reportedly normal value so close to the cutoff may warrant further evaluation, especially in children of parents who are known carriers. Clinicians should maintain a high index of suspicion for CF when infants present with pancreatic insufficiency, severe edematous malnutrition, and/or persistent respiratory infections, or when both parents are CF carriers, even in the setting of a negative newborn screen. Any child with symptoms consistent with CF should undergo diagnostic sweat chloride testing and subsequent genetic testing, if indicated.

## Data Availability

The original contributions presented in the study are included in the article/Supplementary Material, further inquiries can be directed to the corresponding author.

## References

[1] Hoch H Sontag MK Scarbro S Juarez-Colunga E McLean C Kempe A Clinical outcomes in U.S. Infants with cystic fibrosis from 2001 to 2012. Pediatr Pulmonol. (2018) 53(11):1492–7. 10.1002/ppul.24165 30259702

[2] Sontag MK Miller JI McKasson S Gaviglio A Martiniano SL West R Newborn screening for cystic fibrosis: a qualitative study of successes and challenges from universal screening in the United States. Int J Neonatal Screen. (2022) 8(3):1–14. 10.3390/ijns8030038 PMC9326751 35892468

[3] Coverstone AM Ferkol TW. Early diagnosis and intervention in cystic fibrosis: imagining the unimaginable. Front Pediatr. (2020) 8:608821. 10.3389/fped.2020.608821 33505947 PMC7830672

[4] Tridello G Castellani C Meneghelli I Tamanini A Assael BM. Early diagnosis from newborn screening maximises survival in severe cystic fibrosis. ERJ Open Res. (2018) 4(2):1–8. 10.1183/23120541.00109-2017 PMC5909061 29692998

[5] Dijk FN McKay K Barzi F Gaskin KJ Fitzgerald DA. Improved survival in cystic fibrosis patients diagnosed by newborn screening compared to a historical cohort from the same centre. Arch Dis Child. (2011) 96(12):1118–23. 10.1136/archdischild-2011-300449 21994242

[6] Accurso FJ Sontag MK Wagener JS. Complications associated with symptomatic diagnosis in infants with cystic fibrosis. J Pediatr. (2005) 147(3 Suppl):S37–41. 10.1016/j.jpeds.2005.08.034 16202780

[7] Coffey MJ Whitaker V Gentin N Junek R Shalhoub C Nightingale S Differences in outcomes between early and late diagnosis of cystic fibrosis in the newborn screening era. J Pediatr. (2017) 181:137–45.e1. 10.1016/j.jpeds.2016.10.045 27837951

[8] Ross LF. Newborn screening for cystic fibrosis: a lesson in public health disparities. J Pediatr. (2008) 153(3):308–13. 10.1016/j.jpeds.2008.04.061 18718257 PMC2569148

[9] Martiniano SL Hoppe JE Sagel SD Zemanick ET. Advances in the diagnosis and treatment of cystic fibrosis. Adv Pediatr. (2014) 61(1):225–43. 10.1016/j.yapd.2014.03.002 25037130

[10] American College of Medical Genetics and Genomics. ACT sheets and algorithms 2006. Available at: https://www.acmg.net/ACMG/Medical-Genetics-Practice-Resources/ACT_Sheets_and_Algorithms.aspx.

[11] Price JF. Newborn screening for cystic fibrosis: do we need a second IRT? Arch Dis Child. (2006) 91(3):209–10. 10.1136/adc.2005.085084 16492882 PMC2065957

[12] The New York-Mid-Atlantic Consortium for Genetic Newborn Screening Services. Genetic alliance monographs and guides. In: Lisa Wise MA, editor. Understanding genetics: A New York, mid-atlantic guide for patients and health professionals. Washington (DC): Genetic Alliance (2009). p. 19–22. 23304754

[13] Lumertz MS Rispoli T Rosa KMD Pinto LA. False-negative newborn screening result for immunoreactive trypsinogen: a major problem in children with chronic lung disease. J Bras Pneumol. (2019) 45(3):e20180062. 10.1590/1806-3713/e20180062 31271600 PMC6715039

[14] Farrell PM Kosorok MR Laxova A Shen G Koscik RE Bruns WT Nutritional benefits of neonatal screening for cystic fibrosis. Wisconsin cystic fibrosis neonatal screening study group. N Engl J Med. (1997) 337(14):963–9. 10.1056/NEJM199710023371403 9395429

[15] Scurati-Manzoni E Fossali EF Agostoni C Riva E Simonetti GD Zanolari-Calderari M Electrolyte abnormalities in cystic fibrosis: systematic review of the literature. Pediatr Nephrol. (2014) 29(6):1015–23. 10.1007/s00467-013-2712-4 24326787

[16] Cystic fibrosis foundation patient registry 2021 annual data report Bethesda, Maryland ©2022 Cystic Fibrosis Foundation.

[17] Mei-Zahav M Solomon M Kawamura A Coates A Durie P. Cystic fibrosis presenting as kwashiorkor in a Sri Lankan infant. Arch Dis Child. (2003) 88(8):724–5. 10.1136/adc.88.8.724 12876174 PMC1719610

[18] Sandy NS Nogueira RJN. Nutritional treatment of a young infant with cystic fibrosis presenting with severe kwashiorkor dermatosis. J Trop Pediatr. (2019) 65(6):634–7. 10.1093/tropej/fmz008 30897613

[19] Shajil C Sathishkumar D Chiramel MJ Kumar M Varkki S Rose W Kwashiorkor-like dermatosis: a rare presentation of cystic fibrosis. Clin Exp Dermatol. (2021) 46(1):213–5. 10.1111/ced.14438 32931600

[20] Benjamin O Lappin SL. Kwashiorkor. In: Statpearls. Treasure Island (FL): StatPearls Publishing (2021). Available at: https://www.ncbi.nlm.nih.gov/books/NBK507876/

[21] Bhutta ZA Berkley JA Bandsma RHJ Kerac M Trehan I Briend A. Severe childhood malnutrition. Nat Rev Dis Primers. (2017) 3:17067. 10.1038/nrdp.2017.67 28933421 PMC7004825

[22] O'Regan GM Canny G Irvine AD. ‘Peeling paint’ dermatitis as a presenting sign of cystic fibrosis. J Cyst Fibros. (2006) 5(4):257–9. 10.1016/j.jcf.2006.05.003 16797256

[23] Boos MD Briggs S. Scaly dermatitis and edema in an irritable child. JAMA. (2021) 325(4):393–4. 10.1001/jama.2020.10429 33410860

[24] Roongpisuthipong W Phanachet P Roongpisuthipong C Rajatanavin N. Essential fatty acid deficiency while a patient receiving fat regimen total parenteral nutrition. BMJ Case Rep. (2012) 2012:1–4. 10.1136/bcr.07.2011.4475 PMC3387468 22707694

[25] Crone J Huber WD Eichler I Granditsch G. Acrodermatitis enteropathica-like eruption as the presenting sign of cystic fibrosis–case report and review of the literature. Eur J Pediatr. (2002) 161(9):475–8. 10.1007/s00431-002-0982-0 12200605

[26] Krebs NF. Update on zinc deficiency and excess in clinical pediatric practice. Ann Nutr Metab. (2013) 62(Suppl 1):19–29. 10.1159/000348261 23689110

[27] Martiniano SL Croak K Bonn G Sontag MK Sagel SD. Improving outcomes for Colorado's IRT-IRT-DNA cystic fibrosis newborn screening algorithm by implementing floating cutoffs. Mol Genet Metab. (2021) 134(1-2):65–7. 10.1016/j.ymgme.2021.08.005 34489170

[28] Kay DM Maloney B Hamel R Pearce M DeMartino L McMahon R Screening for cystic fibrosis in New York state: considerations for algorithm improvements. Eur J Pediatr. (2016) 175(2):181–93. 10.1007/s00431-015-2616-3 26293390

